# Diagnostic accuracy of the Oral Aesthetic Subjective Impact Score (OASIS) questionnaire for orthodontic treatment need in Nepal: a hospital-based study

**DOI:** 10.1186/s12903-025-07590-y

**Published:** 2025-12-28

**Authors:** Sushant Pandey, Rajesh Gyawali, Prabhat Ranjan Pokharel, Avinash Chaudhary, Samikshya Sangroula, Sailesh Bhattarai

**Affiliations:** 1 Department of Orthodontics and Dentofacial Orthopaedics, B.P. Koirala Institute of Health Sciences, Dharan, 56700 Koshi Nepal; 2 School of Public Health and Community Medicine, B.P. Koirala Institute of Health Sciences, Dharan, 56700 Koshi Nepal

**Keywords:** Diagnostic accuracy, IOTN, OASIS, Nepal, Orthodontic treatment need

## Abstract

**Background:**

The Oral Aesthetic Subjective Impact Score (OASIS) is a questionnaire used to assess self-perception of dental aesthetics. This study aimed to assess the diagnostic accuracy of this questionnaire in determining orthodontic treatment need in Nepali patients and to establish an appropriate cut-off score.

**Methods:**

The original OASIS questionnaire was translated and adapted into the Nepali language. A total of 145 participants aged 14–19 years completed the Nepali version of OASIS (OASIS-N) questionnaire and rated their dental aesthetics on a Visual Analogue Scale. Orthodontic treatment need was then evaluated using the Index of Orthodontic Treatment Need-Dental Health Component (IOTN-DHC) and Aesthetic Component (IOTN-AC). Receiver Operating Characteristic (ROC) analysis was done to assess diagnostic accuracy of the OASIS-N against IOTN-DHC, and to identify the optimal cut-off score. Sensitivity, specificity, and predictive values were calculated for different cut-off scores.

**Results:**

OASIS-N demonstrated high internal consistency (Cronbach’s α = 0.88) and good test–retest reliability (ICC = 0.77). It showed good diagnostic accuracy for detecting orthodontic treatment need, with an area under the ROC curve (AUC) of 0.945 (95% CI: 0.911–0.979). A cut-off score of 14.5 provided the best balance of sensitivity (90.4%) and specificity (75.6%) with substantial agreement with IOTN-DHC (κ = 0.660, *p* < 0.001).

**Conclusions:**

OASIS-N demonstrated good diagnostic accuracy for identifying orthodontic treatment need in Nepal. A cut-off score of 14.5 provided the best balance of sensitivity and specificity. Further multi-centre validation in community-based populations is recommended.

**Supplementary Information:**

The online version contains supplementary material available at 10.1186/s12903-025-07590-y.

## Background

Malocclusion has the third highest prevalence among oral conditions, second only to dental caries and periodontal disease [1]. The effect of malocclusion on psychological well-being, self-esteem, and oral health-related quality of life has been well-established [2–4]. Increased self-awareness about dental appearance, particularly among adolescents and young adults, has heightened the demand for orthodontic treatment. Orthodontic treatment need is traditionally assessed by normative indices such as the Index of Orthodontic Treatment Need (IOTN), Index of Complexity, Outcome and Need (ICON), Dental Aesthetic Index (DAI), and Peer Assessment Rating Index (PAR) [5–8]. Although these indices are valuable for quantifying treatment need, they do not fully reflect patient-centred concerns, which are integral to treatment planning [9–11]. In recent years, questionnaires have become increasingly popular in research and clinical practice as complementary tools for assessing oral health-related quality of life. These tools emphasize the patient’s own perception of their oral condition, supplementing the clinical assessment [12].

Mandall et al. [13] proposed an instrument to assess children’s and adolescents’ perception of oral aesthetics. The questionnaire known as the Oral Aesthetic Subjective Impact Score (OASIS) consists of a series of five questions that help assess the degree of concern or disadvantage that children and adolescents feel due to their tooth arrangement (Supplementary Material 1). The questionnaire is scored on a 7-point Likert scale with total score ranging from 5 to 35 points. The score of the aesthetic component of IOTN (IOTN-AC) for each individual is added to the total OASIS score [13]. Thus, the final OASIS score for each individual could vary from 6 to 45 points. The higher the final value, the more likely a greater negative perception of oral aesthetics. The OASIS questionnaire is brief, easy to administer, and widely used for assessing the aesthetic impact of malocclusion. Unlike other aesthetic perception measures, OASIS integrates both subjective and objective components (via IOTN-AC), making it particularly suitable for contexts where rapid screening and resource-efficient assessment are needed [13].

Orthodontic indices are evaluated not only in terms of their reliability and validity but also in terms of diagnostic accuracy, that is, their ability to correctly identify individuals with and without treatment need [14, 15]. Previous studies have demonstrated that indices such as the IOTN, DAI, ICON, and PAR possess strong diagnostic properties [14, 15]. In contrast, the diagnostic accuracy of the OASIS questionnaire has not been systematically assessed. Although its psychometric properties have been examined across different populations [16–23], evidence regarding its ability to discriminate between individuals with and without definite treatment need remains limited. Furthermore, no standardized cut-off score has been validated. Some studies have adopted arbitrary cut-offs such as sample median [16, 17] or a score of 14 [18, 19] to indicate poor self-perception of oral aesthetics and treatment need, but these values lack empirical justification.

We hypothesized that OASIS questionnaire would demonstrate good diagnostic accuracy for identifying normative treatment need, as subjective aesthetic concern tends to increase with malocclusion severity. The present study aimed to assess the diagnostic accuracy of the OASIS questionnaire in determining orthodontic treatment need in a Nepali population and establish a standardized cut-off score.

## Methods

**Fig. 1:**
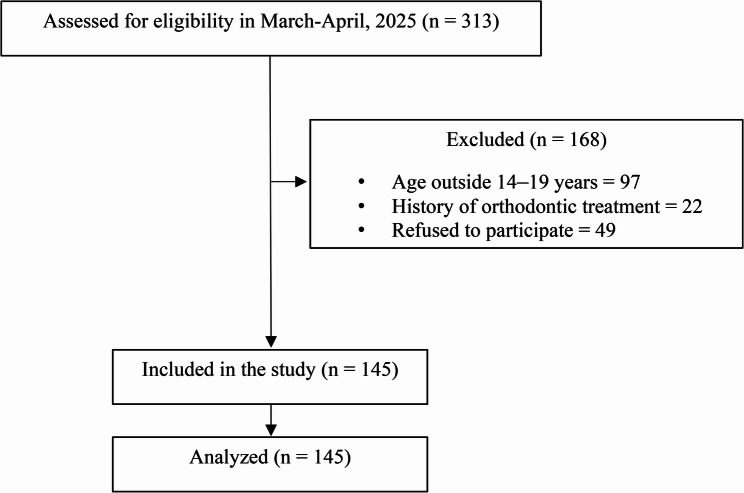
Participant flow chart

This cross-sectional study was conducted in the Department of Orthodontics and Dentofacial Orthopaedics, B.P. Koirala Institute of Health Sciences, Dharan, Nepal, after obtaining ethical clearance from the Institutional Review Committee (IRC/2511/023). Participants were recruited using convenience sampling, with consecutive eligible patients enrolled between March and April 2025. A total of 313 patients were assessed for eligibility; 168 were excluded (97 due to age outside 14–19 years, 22 with a history of orthodontic treatment, and 49 who declined participation), as shown in Fig. 1. The final study sample comprised 145 adolescents aged 14–19 years with no history of orthodontic treatment, orthognathic surgery, maxillofacial trauma, or marked jaw asymmetries. Written informed consent was obtained from all participants and from guardians for those under 18 years.

The Oral Aesthetic Subjective Impact Score (OASIS) was translated into the Nepali language (OASIS-N) following Beaton’s cross-cultural adaptation guidelines [24]. The forward translation in Nepali was carried out independently by two translators, one with a dental background familiar with the study aims and one from a non-medical field. The two versions were reconciled and subsequently back-translated into English by two different bilingual translators who were blinded to the original instrument. An expert committee consisting of the translators and orthodontists reviewed all versions to assess semantic, idiomatic, experiential, and conceptual equivalence and developed a pre-final version. This version was pilot-tested in 30 adolescents to evaluate clarity and comprehensibility, after which minor wording adjustments were made. No major cultural or conceptual issues were identified. Following pilot testing and finalization of the Nepali version, the questionnaire was administered to the main study sample.

A total of 145 participants completed the OASIS-N questionnaire and rated their dental aesthetics on a 100-mm Visual Analogue Scale (VAS), where 0 indicated complete dissatisfaction and 100 indicated complete satisfaction. Orthodontic treatment need was then evaluated using the Index of Orthodontic Treatment Need Dental Health Component (IOTN-DHC) as the normative reference standard, with grades 1–2 categorized as “no/borderline need,” and grades 3–5 as “definite need” [25, 26]. The IOTN-Aesthetic Component (IOTN-AC) was also recorded. The OASIS-N questionnaire was self-administered by participants in a supervised setting. Both the IOTN-AC and the IOTN-DHC were assessed and scored by the principal investigator (SP). Basic literacy was confirmed verbally, and assistance was provided only to clarify instructions when required. The duration of questionnaire completion was not formally recorded. All questionnaires were fully completed, and no missing or incomplete responses were recorded. Blinding between the index test (OASIS-N) and the reference standard (IOTN-DHC) was not possible due to the nature of the data collection process.

Diagnostic accuracy of the OASIS-N for detecting normative treatment need was evaluated using Receiver Operating Characteristic (ROC) analysis, with the IOTN-DHC as the reference standard. The index score used for ROC analysis was the total OASIS score, calculated as the sum of the five OASIS items and the IOTN-AC, in accordance with the original scoring system. The area under the curve (AUC) with 95% confidence intervals was calculated to assess overall accuracy. Sensitivity, specificity, and Youden Index (J) were calculated for each cut-off score. Chi-square test and Cohen’s kappa coefficient (κ) were calculated to evaluate agreement between OASIS-N and IOTN-DHC categories. Positive predictive value (PPV) and negative predictive value (NPV) were estimated at the observed hospital prevalence and additionally modelled at assumed prevalences of 10%, 30%, and 50%. All analyses were performed using raw total OASIS-N scores.

Internal consistency of the translated version was evaluated using Cronbach’s alpha calculated from the pilot test sample of 30 participants. Test–retest reliability was assessed with Intraclass Correlation Coefficient (ICC) in 20 participants after 2 weeks interval using a two-way mixed-effects model ICC(3,1). Normality of all variables was assessed using histograms, Q–Q plots, box plots, and skewness–kurtosis statistics. All variables demonstrated approximate normal distribution. Independent t-tests were used to compare OASIS-N score and VAS score between males and females. Because IOTN-DHC and IOTN-AC are ordinal scales, non-parametric Mann–Whitney U tests were used for group comparisons regardless of distributional appearance. Correlations among OASIS-N, IOTN-DHC, IOTN-AC, and VAS were examined using Spearman’s rank correlation. Statistical analyses were performed using SPSS v26.0 (IBM Corp., Armonk, NY) with significance set as *p* < 0.05.

## Results

The Nepali version of the OASIS questionnaire (OASIS-N) (Supplementary Material 2) demonstrated high internal consistency (Cronbach’s α = 0.88) calculated from the pilot test sample of 30 participants. Test–retest reliability was good, with ICC(3,1) = 0.77 (95% CI: 0.50–0.90) based on 20 participants retested after 2 weeks. A total of 145 participants completed the questionnaire. The mean OASIS-N score was 21.28 ± 9.46 and the mean VAS score regarding self-perception of aesthetics was 48.25 ± 26.7 (Table 1). The median IOTN-DHC score was 3 (IQR: 2–4), with 71.7% of participants classified as having definite treatment need (grades 3–5).

**Table 1:** Demographic and clinical characteristics of the participants

Variables	Male (*n* = 66)	Female (*n* = 79)	Total (*n* = 145)	*p*-value	Effect size
Age (Mean ± SD)	16.74 ± 1.49	16.53 ± 1.7	16.63 ± 1.6	0.433	*d* = 0.13
IOTN-DHC (Median, IQR)	3(2–4)	3(2–4)	3 (2–4)	0.042*	*r* = 0.19
IOTN-AC (Median, IQR)	4(3–5)	5(3–6)	5 (3–6)	0.020*	*r* = 0.22
OASIS-N score (Mean ± SD)	18.79 ± 8.67	23.37 ± 9.64	21.28 ± 9.46	0.003*	*d* = -0.50
VAS score (Mean ± SD)	53.38 ± 25.35	43.96 ± 27.25	48.25 ± 26.7	0.034*	*d* = 0.36

*SD* Standard Deviation, *IQR* Interquartile Range, *d* Cohen’s d, *r* Rank-biserial r, *p < 0.05 indicates statistical significance

**Fig. 2:**
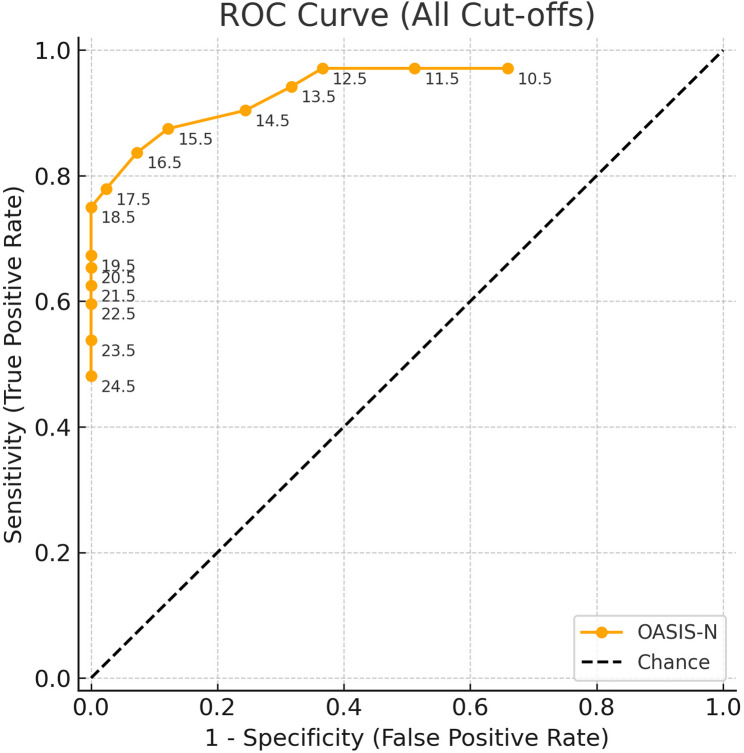
ROC curve for the OASIS-N showing all tested cut-offs (10.5–24.5). Area under the curve (AUC) = 0.945 (95% CI, 0.911–0.979)

**Fig. 3:**
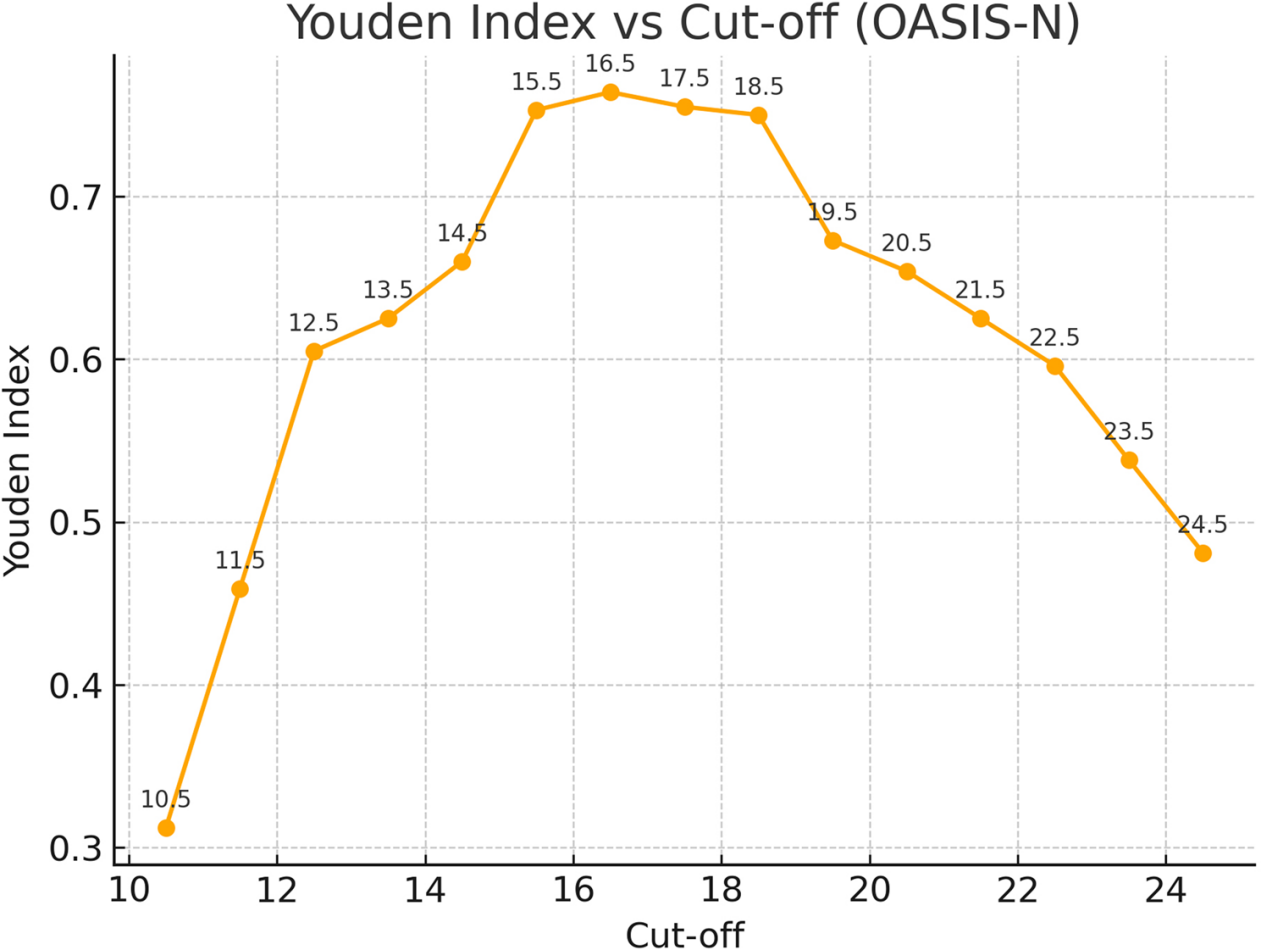
Youden index across OASIS-N cut-off scores, showing a plateau between 15.5 and 18.5 with the maximum at 16.5

Receiver Operating Characteristic (ROC) curve analysis demonstrated good diagnostic accuracy of OASIS-N for detecting normative treatment need (AUC = 0.945; 95% CI: 0.911–0.979, *p* < 0.001) (Fig. 2). The Youden Index curve (Fig. 3) demonstrated a plateau of high diagnostic performance between cut-offs of 15.5 and 18.5, with the maximum value observed at 16.5. Decimal thresholds result from ROC analysis, which identifies optimal cut-points lying between integer score values. Operationally, a threshold of 14.5 indicates that individuals with scores ≥ 15 are classified as having definite treatment need.

At a cut-off score of 14.5, OASIS-N achieved a sensitivity of 90.4% and a specificity of 75.6%, with a Cohen’s kappa of 0.660 (*p* < 0.001) (Table 2). At this cut-off, out of 145 participants, OASIS-N correctly classified 94 true positives and 31 true negatives, with 10 false positives and 10 false negatives (Table 3). Chi-square analysis further confirmed significant agreement between OASIS-N cutoff of 14.5 and IOTN-DHC categories (χ²=63.151, *p* < 0.001). At the observed hospital prevalence of 71.7%, the positive predictive value (PPV) was 0.904 and the negative predictive value (NPV) was 0.756. PPV and NPV modelled at assumed prevalences of 10%, 30%, and 50% demonstrated increasing PPV and decreasing NPV with higher prevalence (Table 4).

**Table 2:** Diagnostic accuracy of OASIS-N at different cut-off for identifying orthodontic treatment need (Prevalence = 71.7%)

Cut-off	Sensitivity% (95% CI)	Specificity% (95% CI)	PPV (95% CI)	NPV (95% CI)	Youden Index (J)	Cohen’s κ
13.5	94.2 (87.9–97.3)	68.3 (53.0–80.4)	0.883 (0.808–0.936)	0.824 (0.655–0.932)	0.625	0.636
14.5	90.4 (83.2–94.7)	75.6 (60.6–86.2)	0.904 (0.830–0.953)	0.756 (0.597–0.876)	0.660	0.660
15.5	87.5 (79.8–92.5)	87.8 (74.4–94.7)	0.948 (0.883–0.983)	0.735 (0.589–0.851)	0.753	0.711
16.5	83.8 (75.4–89.6)	92.7 (80.6–97.5)	0.967 (0.906–0.993)	0.691 (0.552–0.809)	0.764	0.693
17.5	77.9 (69.0–84.8)	97.6 (87.5–99.6)	0.988 (0.934–0.999)	0.635 (0.504–0.753)	0.755	0.658

*CI* Confidence Interval, *PPV* Positive Predictive Value, *NPV* Negative Predictive Value

**Table 3:** Cross-tabulation of OASIS-N cut-off 14.5 and orthodontic treatment need

	IOTN-DHC Grades 3–5 (Definite treatment need)	IOTN-DHC Grades 1–2 (No/Little treatment need)	Total
OASIS-*N* ≥ 14.5	94 (True Positive)	10 (False Positive)	104
OASIS-*N* < 14.5	10 (False Negative)	31 (True Negative)	41
Total	104	41	145

*IOTN-DHC* Index of Orthodontic Treatment Need Dental Health Component

**Table 4:** Modeled positive predictive value (PPV) and negative predictive value (NPV) of OASIS-N at cut-offs 13.5–17.5 across assumed prevalences of 10%, 30%, and 50%

Cut-off	Prevalence	Sensitivity	Specificity	PPV (95% CI)	NPV (95% CI)
13.5	10%	0.942	0.683	0.262 (0.199–0.330)	0.985 (0.978–0.990)
13.5	30%	0.942	0.683	0.556 (0.491–0.618)	0.934 (0.914–0.951)
13.5	50%	0.942	0.683	0.748 (0.693–0.797)	0.885 (0.854–0.912)
14.5	10%	0.904	0.756	0.295 (0.227–0.370)	0.983 (0.975–0.990)
14.5	30%	0.904	0.756	0.606 (0.540–0.667)	0.922 (0.901–0.942)
14.5	50%	0.904	0.756	0.786 (0.739–0.830)	0.865 (0.832–0.894)
15.5	10%	0.875	0.878	0.455 (0.358–0.534)	0.985 (0.977–0.991)
15.5	30%	0.875	0.878	0.736 (0.668–0.795)	0.925 (0.903–0.946)
15.5	50%	0.875	0.878	0.884 (0.841–0.920)	0.874 (0.841–0.903)
16.5	10%	0.837	0.927	0.645 (0.536–0.748)	0.973 (0.962–0.982)
16.5	30%	0.837	0.927	0.857 (0.799–0.909)	0.886 (0.860–0.909)
16.5	50%	0.837	0.927	0.940 (0.907–0.965)	0.810 (0.770–0.846)
17.5	10%	0.779	0.976	0.757 (0.644–0.852)	0.967 (0.954–0.979)
17.5	30%	0.779	0.976	0.898 (0.843–0.941)	0.857 (0.827–0.886)
17.5	50%	0.779	0.976	0.948 (0.914–0.972)	0.769 (0.729–0.806)

*PPV* Positive Predictive Value, *NPV* Negative Predictive Value, *CI* Confidence Interval

Both the mean OASIS-N score and the mean VAS score were significantly higher in females (*p* < 0.05). OASIS-N showed strong positive correlations with IOTN-DHC (r_s_ = 0.86, *p* < 0.001) and IOTN-AC (r_s_ = 0.82, *p* < 0.001). A strong negative correlation was observed between OASIS-N and VAS scores (*r* = − 0.88, *p* < 0.001).

## Discussion

The present study demonstrated that the Nepali version of the Oral Aesthetic Subjective Impact Score (OASIS-N) is a reliable and valid tool for assessing self-perception of dental aesthetics, with good diagnostic accuracy in identifying orthodontic treatment need. A cut-off score of 14.5 achieved a favorable balance between sensitivity (90.4%) and specificity (75.6%), with substantial agreement with the IOTN-DHC, within the study population. These findings suggest that OASIS-N can serve as a screening tool for orthodontic treatment need, complementing normative indices.

Receiver Operating Characteristic (ROC) analysis returned an area under the curve (AUC) of 0.945. Comparable values close to 0.95 have been reported for indices such as the DAI, and IOTN, whereas slightly lower values between 0.82 and 0.85 have been reported for indices like ICON and PAR [14, 15]. The high AUC observed for OASIS-N in our study should be interpreted with caution as this was a hospital-based study which included many participants actively seeking orthodontic treatment.

Cut-off selection is critical for balancing sensitivity and specificity depending on the intended application [27]. Lower thresholds may enhance sensitivity but risk classifying more individuals as treatment candidates [14]. The optimal threshold should reflect not only diagnostic accuracy but also the clinical and social implications of false positive or false negative classification [15]. In our study, the Youden Index plot (Fig. 3) demonstrated a plateau from 15.5 to 18.5, indicating that multiple thresholds in this range achieve high overall accuracy. Although the maximum Youden Index value occurred at 16.5, this threshold produced a marked reduction in sensitivity (83.2%). Similarly, a cut-off of 15.5 improved specificity but the sensitivity remained below 90% (Table 2). Because OASIS-N is intended for preliminary screening rather than definitive diagnosis, a threshold that retains higher sensitivity is preferable to minimize false negatives.

At a threshold of ≥ 14.5 points, OASIS-N achieved 90.4% sensitivity and 75.6% specificity, with substantial agreement with IOTN-DHC classification (κ = 0.660), providing a favourable balance between sensitivity and specificity. Lowering the cut-off to 13.5 increased sensitivity (94.2%) and NPV (0.823) marginally, but reduced specificity (68.3%) and PPV (0.883) leading to a higher rate of false positives. Conversely, higher cut-offs (15.5–17.5) improved specificity (0.878–0.976) and PPV (0.948–0.988), but sensitivity dropped below 90% (0.875–0.779) and NPV decreased (0.735–0.635) (Table 2). Earlier studies did not propose diagnostic cut-offs, but used thresholds such as 14 or the sample median for analysis [16–19]. Our empirically derived cut-off (≥ 14.5) falls within this range. Differences across studies may reflect cultural aesthetic norms, sample severity, and the clinical settings in which the studies were conducted.

At the observed hospital prevalence of 71.7%, PPV was high (0.904) and NPV was modest (0.756). This distribution reflects the case-mix of a tertiary referral setting, where most patients already present with significant malocclusion. Predictive values are prevalence-dependent. PPV and NPV were modelled at assumed prevalences of 10%, 30%, and 50%. This confirmed the expected pattern: as prevalence increases, PPV rises while NPV falls. This finding underscores the need to interpret predictive values in the context of the population under study. In community-based surveys, where the prevalence of definite treatment need is likely lower than in hospitals, PPV would decrease and NPV would increase. Thus, OASIS-N may be more effective for ruling out treatment need rather than confirming it in large-scale screening programs such as school-based initiatives.

The diagnostic accuracy estimates of this study should be interpreted in the context of potential spectrum bias [28]. Because the sample was drawn from a hospital-based population, the distribution of malocclusion severity was disproportionately weighted toward moderate and severe cases, yielding a high prevalence of definite treatment need (71.7%). This enriched severity spectrum can artificially elevate PPV and may also lead to higher AUC values than would be expected in community-based samples with a broader range of malocclusion severity [28]. The distribution of IOTN-DHC grades for the study sample is provided in Supplementary Material 3 to illustrate the severity spectrum.

The internal consistency of OASIS-N (α = 0.88) was higher than that reported in the Brazilian version (α = 0.52) [29] and comparable to the Farsi version (α = 0.87) [30]. The mean OASIS-N score (21.28 ± 9.46) was comparable to findings from India (23.29) [31] but higher than those reported in Malaysia (15.07 ± 5.05) [19]. Variation in internal consistency and mean OASIS scores is likely influenced by a combination of cultural, methodological, and contextual factors. Cultural norms and societal expectations regarding dental aesthetics influence how individuals perceive malocclusion [32]. Differences arising from translation and cross-cultural adaptation procedures may also introduce subtle semantic or conceptual shifts that affect item interpretation, even when rigorous guidelines are followed [33]. In addition, socioeconomic context, including awareness of orthodontic treatment and perceived affordability, has been shown to influence self-perceived treatment need and may contribute to the variability observed across populations [34, 35].

Females showed higher IOTN and OASIS scores than males, consistent with studies in other populations [19, 30], likely reflecting greater aesthetic self-awareness. The mean VAS score (48.25 ± 26.7) was also comparable to that reported in another study (40.16 ± 18.1) [36]. Strong correlations between OASIS-N and both IOTN-DHC (r_s_ = 0.86) and IOTN-AC (r_s_ = 0.82) confirm its construct validity which is in agreement with previous reports [22, 37], though some studies have shown weaker associations [26, 38]. The strong negative correlation with VAS (*r* = − 0.88) further supports validity, consistent with findings from other studies [31, 36].

The strong association of OASIS-N with IOTN-AC is expected, as both instruments assess perceived dental aesthetics and OASIS-N incorporates the IOTN-AC score within its total scoring. This creates partial non-independence and shared method variance between the two measures, contributing to their high correlation. However, a previous study, in which the IOTN-AC score was not incorporated into the total OASIS score, reported a weak correlation between OASIS and IOTN-AC [38]. The strong correlation of OASIS-N with IOTN-DHC likely reflects the severity distribution within our hospital-based sample, where a high proportion of participants presented with moderate-to-severe malocclusion, increasing alignment between subjective concern and normative clinical need. These contextual factors indicate that the magnitude of these correlations should be interpreted with caution.

From a clinical perspective, OASIS-N offers several advantages. It is quick to administer, requires no specialized equipment, and captures both subjective and objective elements of dental aesthetics by incorporating IOTN-AC into its scoring. While indices like IOTN-DHC remain the gold standard for normative assessment, OASIS-N’s diagnostic accuracy suggests it could be a valuable initial screening tool in community surveys, tele-dentistry applications, and primary dental care, reducing the burden on orthodontic specialists. Its brief, self-administered format also makes it feasible for use in school-based or outreach screening, and digital versions could be integrated into mobile or web platforms to support remote self-assessment and triage. However, a separate, population-based cut-off would be required for community screening, as the 14.5 threshold identified in this hospital-based sample is not directly generalizable.

Some limitations of this study must be acknowledged. The study sample was convenient and hospital-based and therefore characterized by a high prevalence of treatment need, which may not reflect the prevalence in the general population of Nepal. The lack of blinding between the index test and reference standard introduces the possibility of examiner-related bias, which may have inflated correlations and kappa agreement. In addition, a formal content validity assessment using the Content Validity Index (CVI) or Content Validity Ratio (CVR) was not performed. Future studies should include population-based sampling, diverse demographic groups, multi-centric designs, and a formal content validity assessment to strengthen generalizability.

## Conclusion

The Nepali version of the OASIS (OASIS-N) demonstrated good diagnostic accuracy for identifying orthodontic treatment need in this hospital-based sample. A cut-off score of 14.5 provided the best balance of sensitivity and specificity. Further multi-centre validation in community-based populations is recommended to strengthen generalisability.

## Supplementary Information

Supplementary Material 1. Oral Aesthetic Subjective Impact Score (OASIS) questionnaire.

Supplementary Material 2. Nepali version of the OASIS questionnaire (OASIS-N).

Supplementary Material 3. Distribution of treatment need of the participants based on the Index of Orthodontic Treatment Need – Dental Health Component (IOTN-DHC).

## Data Availability

The data underlying this article will be shared on reasonable request to the corresponding author.

## Abbreviations

AUC: Area Under the Curve
CVI: Content Validity Index
CVR: Content Validity Ratio
DAI: Dental Aesthetic Index
ICC: Intraclass Correlation Coefficient
ICON: Index of Complexity, Outcome and Need
IOTN: Index of Orthodontic Treatment Need
IOTN-AC: Index of Orthodontic Treatment Need – Aesthetic Component
IOTN-DHC: Index of Orthodontic Treatment Need – Dental Health Component
IQR: Interquartile Range
NPV: Negative Predictive Value
OASIS: Oral Aesthetic Subjective Impact Score
OASIS-N: Nepali Version of the Oral Aesthetic Subjective Impact Score
PAR: Peer Assessment Rating Index
PPV: Positive Predictive Value
ROC: Receiver Operating Characteristic
SD: Standard Deviation
SPSS: Statistical Package for the Social Sciences
VAS: Visual Analogue Scale
