# Supplementary material for: Diagnostic accuracy of the Oral Aesthetic Subjective Impact Score (OASIS) questionnaire for orthodontic treatment need in Nepal: a hospital-based study
Source: BMC Oral Health. 2025 Dec 28;26:198. doi: 10.1186/s12903-025-07590-y (PMC12859989; doi:10.1186/s12903-025-07590-y)
Supplement: Supplementary file 1 — Supplementary Material 1. Oral Aesthetic Subjective Impact Score (OASIS) questionnaire. [file 12903_2025_7590_MOESM1_ESM.pdf]

Oral Aesthetic Subjective Impact Score (OASIS) questionnaire

How do you feel about the appearance of your teeth?

	1	2	3	4	5	6	7	
Not concerned at all								Very concerned

Have you found that other people have commented on the appearance of your teeth?

	1	2	3	4	5	6	7	
Not at all								All the time

Have you found that other people have teased you about the appearance of your teeth?

	1	2	3	4	5	6	7	
Not at all								All the time

Do you try to avoid smiling because of the appearance of your teeth?

	1	2	3	4	5	6	7	
Not at all								All the time

Do you ever cover your mouth because of the appearance of your teeth?

	1	2	3	4	5	6	7	
Not at all								All the time
