# Supplementary material for: Diagnostic accuracy of the Oral Aesthetic Subjective Impact Score (OASIS) questionnaire for orthodontic treatment need in Nepal: a hospital-based study
Source: BMC Oral Health. 2025 Dec 28;26:198. doi: 10.1186/s12903-025-07590-y (PMC12859989; doi:10.1186/s12903-025-07590-y)
Supplement: Supplementary file 2 — Supplementary Material 2. Nepali version of the OASIS questionnaire (OASIS-N). [file 12903_2025_7590_MOESM2_ESM.pdf]

Nepali version of the OASIS questionnaire (OASIS-N)

१. तपाईलाई आफ्नो दाँत देखेर कस्तो लाग्छ ?

१ २ ३ ४ ५ ६ ७

अलिकति पनि चासो राख्दिन धेरै चासो राख्दछु

२. तपाईको दाँतलाई लिएर अरुले कुरा गरेको पाउनुभएको छ ?

१ २ ३ ४ ५ ६ ७

अहिले सम्म छैन सधैं भरि

३. तपाईको दाँतलाई लिएर अरुले जिस्काउने गरेको अनुभव छ ?

१ २ ३ ४ ५ ६ ७

अहिले सम्म छैन सधैं भरि

४. के तपाईले आफ्नो दाँत नदेखियोस भनेर हाँसो रोक्ने प्रयास गर्नुभएको छ ?

१ २ ३ ४ ५ ६ ७

अहिले सम्म छैन सधैं भरि

५. के तपाईको आफ्नो दाँत देखिन्छ भनेर छोप्ने गर्नुभएको छ ?

१ २ ३ ४ ५ ६ ७

अहिले सम्म छैन सधैं भरि
