# Supplementary material for: Diagnostic accuracy of the Oral Aesthetic Subjective Impact Score (OASIS) questionnaire for orthodontic treatment need in Nepal: a hospital-based study
Source: BMC Oral Health. 2025 Dec 28;26:198. doi: 10.1186/s12903-025-07590-y (PMC12859989; doi:10.1186/s12903-025-07590-y)
Supplement: Supplementary file 3 — Supplementary Material 3. Distribution of treatment need of the participants based on the Index of Orthodontic Treatment Need – Dental Health Component (IOTN-DHC). [file 12903_2025_7590_MOESM3_ESM.docx]

Supplementary table 1. Distribution of treatment need of the participants based on the Index of Orthodontic Treatment Need – Dental Health Component (IOTN-DHC)

| **IOTN-DHC grading** | **N (%)** |
| --- | --- |
| 1 - No treatment need | 5 (3.4%) |
| 2 - Mild/Little treatment need | 36 (24.8%) |
| 3 - Moderate/Borderline treatment need | 44 (30.3%) |
| 4 - Severe/Great treatment need | 39 (26.9%) |
| 5 - Extreme/Very great treatment need | 21 (14.5%) |
| Total | 145 (100%) |
